# The Haitian Orthopaedic Residency Exchange Program

**DOI:** 10.5435/JAAOSGlobal-D-19-00027

**Published:** 2019-08-06

**Authors:** Robert Belding, Gregory Grabowski, Kevin Williams, Kyle Mobley, Chris Bray, Shane Woolf, Patrick Jumelle, David Koon

**Affiliations:** From Palmetto Health-USC Orthopedic Center, Columbia, SC (Dr. Grabowski, Dr. Williams, Dr. Mobley, and Dr. Koon); the Department of Orthopaedic Surgery, Greenville Health, System, Greenville, SC (Dr. Bray); the Department of Orthopaedics and Physical Medicine, Medical University of South Carolina, Charleston, SC (Dr. Woolf); and the Department of Orthopaedic Surgery, Hospital de la Paix, Port au Prince, Haiti (Dr. Jumelle).

## Abstract

After a devastating earthquake in Haiti in 2010, multiple South Carolinian orthopaedic teams, funded by the South Carolina Orthopedic Association (SCOA), developed an exchange program for Haitian residents.

**Methods:**

SCOA teams have sequentially logged their patient experiences since 2015 for a total of six updates per year. These logs were reviewed in detail to evaluate clinical results in terms of case volumes, cases performed, follow-up obtained, and complications.

**Results:**

Twenty-one orthopaedic attendings, 19 South Carolina orthopaedic residents, 22 Haitian orthopaedic residents, and 22 ancillary staff have rotated through Hospital Lumiere. The teams have seen over 2000 patients in the orthopaedic clinic and performed 554 surgeries, including 207 fractures (half of which being open), 24 nonunion and 7 malunion repairs, 15 lower extremity amputations, 27 hemiarthroplasties for femoral neck fractures, and 34 cases of chronic osteomyelitis.

**Discussion:**

The SCOA Foundation has developed a coordinated service for the musculoskeletal needs of the Haitian people while collaboratively elevating the standard of orthopaedic training in Haiti. We report a collaborative model that other US residency programs can use to impart beneficial changes not only in their home program, but also in training programs abroad.

On January 12, 2010, at 4:53 pm, a catastrophic earthquake struck the small island nation of Haiti. The epicenter of the magnitude 7.0 Mw earthquake was near the town of Leogane and could be felt as far away as Cuba, Jamaica, Venezuela, and Puerto Rico. The death toll from the earthquake has been reported as high as 160,000 lives and associated infrastructural damage was substantial. Almost one million people were displaced from homes and families. There is no reliable estimate for the number of Haitians injured. Unbeknownst to most of us, Drs. Robert Belding and Rick Reed, two orthopaedic surgeons from South Carolina (SC), mobilized and immediately set out for Haiti along with three emergency medical technicians by a small private aircraft. On January 15, they arrived at a small mountain missionary medical facility called Hospital Lumiere, which is located about 90 miles from the epicenter of the earthquake in Bonne Fin, Haiti. Before their arrival, most of the medical staff at the hospital had fled to Port-au-Prince to find their families while over 250 patients had made their way to Hospital Lumiere, which has a capacity of about 125 med-surg beds. Triage and stabilization of patients was the first order of business. The small team encountered crush injuries, fractures, open wounds, partial amputations, and a wide variety of other orthopaedic injuries. Over the next several days they cared for scores of patients, sometimes working through the night.

During the next 4 months, five additional teams from SC, coordinated through the Medical University of South Carolina (MUSC) Department of Orthopaedics, alternated visits with other volunteer medical teams, including teams organized by Apostolic Christian World Relief (ACWR), to treat, manage, and rehabilitate the injured at Hospital Lumiere. The consequences of the earthquake underscored the need for modern wound management techniques and coordinated medical and surgical care of the injured and postoperative patients, as well as a critical need to help educate and grow the Haitian orthopaedic surgeon network. It was imperative to provide continuity of care, to elevate the standard of care, and also to impart knowledge and skills to the gracious Haitian orthopaedic surgeons, who are woefully outnumbered and overstretched with respect to the country's population. Furthermore, the consequences of orthopaedic injury are severe in Haiti, where the terrain is rugged, daily life is demanding for everyone, and comprehensive medical care is lacking, if available at all. Simply put, people wither or die from injuries and conditions that would be readily treatable in most sovereign nations.

The relationship between Hospital Lumiere and SC orthopaedic surgeons had its origin decades earlier. In 1996, at the request of Dr. Jim Augustine (Professor Emeritus, University of South Carolina [USC] School of Medicine), advisor to Christian Medical & Dental Society, Dr. Robert Belding took a group of five medical students to Hospital Lumiere during their spring break for a Christian medical mission experience. Dr. Belding continued to do this spring break trip for 5 years, making additional trips to the hospital with work teams, medical specialists, and orthopaedic residents. Dr. Joseph D. Thompson, an orthopaedic surgeon from Charleston, SC, organized and financed one MUSC resident per year to spend a week with the team at Hospital Lumiere. From 1996 to 2009, Dr. Belding taught Haitian medical students and orthopaedic residents from both Haiti and SC at Hospital Lumiere, eventually spending 1 week a month at the hospital himself. From the late 1990s until today, Hospital Lumiere has received orthopaedic surgical equipment donated by various hospitals, medical offices, and orthopaedic equipment representatives in SC. An estimated 5.5 million dollars in used or surplus equipment, supplies, and implants have been shipped to the hospital over the years. This donated material allows the teams to treat patients who may or may not be able to afford their care otherwise.

Having witnessed the dire need for more formal, organized Haitian orthopaedic training and from his experience with the MUSC and USC programs in SC, Dr. Belding envisioned a coordinated and collaborative international educational exchange program. His vision was to have scheduled teams from SC, including faculty and a resident, travel to Hospital University de la Paix, teach on rounds and conference to the residents in Port, and then travel four hours along with a senior and junior Haitian resident to Hospital Lumiere to spend the remainder of the week, providing orthopaedic clinical and surgical care. He reached out to the leadership of the three SC orthopaedic residency programs (MUSC, USC, and the Greenville Health System program) as well as the residency program director at HUP in Port au Prince and Haitian national orthopaedic leadership. Some of the many people engaged in the early vision for the residency exchange program included Dr. Patrick Jumelle (Residency Program Director, Hospital de la Paix), Dr. Jean Mary Fritz Henry (Department Chair, Hospital de la Paix), Dr. Hans Larsen (Haitian Orthopaedic and Traumatology Association), Dr. Kay Wilkins (AAOS Haiti Consultant), the leadership of ACWR and Hospital Lumiere, and the three SC orthopaedic residency program stakeholders.

Dr. Belding and colleagues needed a central organization to help fund, recruit, coordinate, and oversee the exchange program to ensure viability. The South Carolina Orthopaedic Association (SCOA) was approached for this purpose. The Association's leadership agreed to work with Belding and the three residency programs to take on this role. SCOA created a 501(c) (3) foundation to support the SC Global Orthopaedic Resident Education Initiative, which was approved at its annual meeting in August of 2014. Lindsey Demos, a physical therapist who had been in Haiti with the MUSC team after the earthquake, took on the role of a coordinator. Since then, SCOA has helped to support the effort financially and administratively, while providing a virtual home for the program through its website (https://www.scoanet.org/foundation.html). The program was endorsed and initially financially supported by the AAOS Board of Councilors, thanks to Dr. Jim O'Leary's efforts.

Because of this collaboration, Haitian residents are exposed to current treatment methods not readily available in other Haitian facilities due to lack of equipment, training, and resources. The American residents have an opportunity to experience a third world culture and provide orthopaedic care in a setting of limited resources to patients with a wide variety of illnesses, including very late presentations of common pathologies. They see the natural course of orthopaedic injuries and diseases when untreated and experience how Haitian physicians manage complications without a wide array of modern equipment. For example, they are taught how to treat femoral neck fractures with an Austin-Moore monoblock prosthesis. This prosthesis was developed and first implanted in 1940 in Columbia, SC, home of one of the participating residency programs (USC).

Additional challenges include those associated with the lack of modern medical technology afforded in most medical centers in America. Residents learn the value of preparing for cases with both fracture planning and anticipating instrument needs for sterilization purposes. Many implants used in the cases were donated from SC organizations; however, most are somewhat antiquated and have been replaced with more modern designs in America. Transporting current equipment presents a constant challenge to keep Haitian hospitals supplied. Fortunately, because of generous donations from SCOA and other organizations, intraoperative fluoroscopy is available for cases, but many other resources commonly used in hospitals in the United States are not available in the austere environment of the Haitian hospitals. These challenging circumstances allow for creative treatment designs and innovative thinking for the trip participants that are unique to this particular experience.

## Methods

Since its inception in the late 2014, the SCOA Foundation has supported and coordinated bimonthly orthopaedic teams from SC beginning in January 2015. The orthopaedic residency program at Hopital Universitaire de la Paix in Port au Prince has been partnering with the SCOA Foundation since its inception. It has provided in-country logistical support and leadership, as well as educational support for residents of both countries. Each orthopaedic residency program in SC is responsible for organizing two teams per year. Therefore, every other month, a team from SC travels to Haiti and is able to perform not only routine clinical care on the immediate population but also follow up care on the previous teams' patients. The team first travels to Port au Prince and performs ward rounds, consultations, and lectures for the Haitian orthopaedic residents at HUP. The following day, the team, along with a junior and senior Haitian orthopaedic resident, travels to Hospital Lumiere, where they serve for the week treating acute and chronic conditions.

A typical team at Lumiere consists of two US attendings, one senior US resident, and two Haitian residents, as well as ancillary US staff of physical therapists, nurses, and/or surgical technicians. One attending staffs the daily orthopaedic clinic with one of the Haitian residents. The Haitian resident presents each patient to the attending. The other US attending provides oversight for surgical cases, with involvement depending on the complexity of the case. Each case involves the senior US resident and at least one Haitian resident scrubbed. This typically follows a dynamic similar to that in most US programs, with the senior US resident walking the Haitian resident through cases as he would at his home program with his own junior residents. In more complicated cases requiring more attending involvement, the senior US resident functions more as a primary surgeon being guided by the attending while the Haitian resident assists. Occasionally, the second US attending will run a second operating room (OR) with only a Haitian resident assisting. For rarer cases, a Haitian attending may also be present with variable direct involvement. We have found excellent camaraderie between US and Haitian residents, which is fostered by their return trip to the US for a week rotation at the home SC program.

In addition to running one or two fully staffed operating rooms, the team conducts daily orthopaedic clinics, provides emergency department consultation, performs morning and afternoon ward rounds, and prepares its own equipment for the next day's surgical cases. This preoperative preparation is key to the efficiency of the team. Assisted by the staff Haitian orthopaedic surgeon at Lumiere, Dr. Mukkuaka Oda, the team reviews the case log for the next day and pulls the necessary surgical equipment for each surgery, selecting instruments and implants to include in trays for sterilization overnight in diesel powered steam autoclaves. Given the limited equipment and resources available, the residents learn that meticulous preoperative planning optimizes outcomes. At the conclusion of the workweek, the team performs ward rounds with Dr. Oda and the clinical care coordinator to assure appropriate transition of care. In addition, Belding's vision included having Haitian residents visit one of the SC orthopaedic training programs for an observership three times per year. This component has enabled the visiting residents to see the way American orthopaedic training is structured, as they are able to attend orthopaedic educational lectures and journal clubs, observe on-call consultations, outpatient clinics, and outpatient and inpatient surgical cases. This has resulted in the Haitian residents elevating their own core competencies of professionalism, practice-based learning, patient care and clinical skills, medical knowledge, systems-based practice, and interpersonal skills.

**Figure 1:**
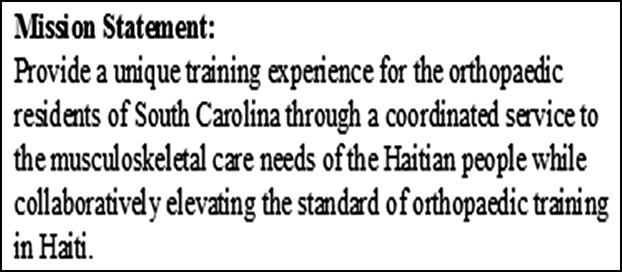
Mission statement.

The SCOA Foundation covers resident expenses, and the international elective is considered a component of the orthopaedic trauma rotation at each of the three US residency programs. Thus, residents are held to Accreditation Council of Graduate Medical Education standards in compliance with their training (Figure 1).

## Results and Statistics

When the SCOA program was started, there were only 66 orthopaedic surgeons in total practicing in the nation of nearly 11 million and land area about 1/3 that of SC. There are now about 128 Haitian orthopaedic surgeons, still far below a suitable number to support the national need. Primary care and other traditional medical specialties are also understaffed. Access to any form of Western medical care is often challenging, if not impossible. This is especially true in the remote areas of the northern and southern peninsulas. Occasionally, treatment by the local witch doctor further complicates patient care. Given the relative lack of local orthopaedic care, the teams frequently see the natural outcomes of orthopaedic maladies.

The Haitian medical record system revolves around paper hospital charting and small cards that are kept by the patients themselves. Both the ancillary staff at the hospital as well as the residents and attending physicians rounding for the week update these records. In addition, the SCOA foundation leadership recognized the need to ensure high-quality longitudinal care, continuity, assessment of efficacy and results, and ownership of perioperative complications similar to standards back home. For quality assurance, a secure follow-up record was created and shared among providers for those patients undergoing orthopaedic treatment. The log includes both surgical and nonsurgical cases undertaken in Haiti by the treatment team, allows the teams to communicate regarding patient outcomes, and allows for recording and proper management of any complications that might occur. Since 2015 this log has sequentially been updated by each visiting team for a total of six updates per year. These records help establish a quality metric to determine surgical case volume and complication rate as determined by our senior operating orthopaedic surgeons. This in turn has helped the hospital administration at Lumiere to determine staffing and resource needs and helps the SCOA program confirm patient safety and quality of care. We are able to report on hospital quality assurance data for case volumes, complications, and case types.

**Figure 2:**
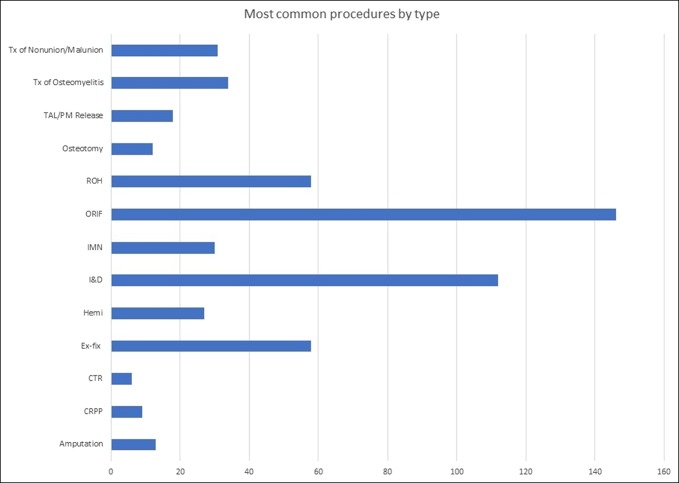
Number of cases by procedure type.Tx = Treatment; TAL = Tend-Achilles Lengthening; PM = Posteromedial; ROH = Removal of Hardware; ORIF = Open Reduction Internal Fixation; IMN = Intramedullary Nail; I&D = Irrigation and Debridement; Hemi = Hemiarthroplasty; Ex-fix = External Fixator; CTR = Carpal Tunnel Release; CRPP = Closed Reduction Percutaneous Pinning.

Over the 4 years since program inception, 21 orthopaedic attendings have supervised teams, 19 SC orthopaedic residents have participated, and 22 Haitian orthopaedic residents have rotated through Hospital Lumiere. In addition, 22 ancillary staff members accompanied teams to Haiti. The outcomes that have been achieved by this program are no less than outstanding. To date, the teams have seen over 2000 patients in the orthopaedic clinic, supervised over 650 inpatients, and performed 554 surgeries. The surgical diversity is quite remarkable, especially given the fact that many traumatic injuries present to the clinic weeks to months after the initial injury. The teams have specifically concentrated on teaching basic orthopaedic principles and have avoided equipment-dependent interventions like total joint arthroplasty, arthroscopy, and spinal surgeries. Acute fractures, malunions, nonunions, acute and chronic infections, untreated congenital abnormalities, and soft-tissue and osseous tumors are most commonly encountered. The teams have treated 207 fractures (with over 100 of these being open injuries), 24 nonunion and 7 malunion repairs, 15 lower extremity amputations, and 27 femoral neck fractures necessitating hemiarthroplasty. In addition, 12 osteotomies were performed, along with carpal tunnel releases, posteromedial hindfoot releases, and tendoachilles lengthenings. Thirty-four patients were treated for chronic osteomyelitis (Figure 2).

To date, in clean cases, one patient developed an infection that responded to oral antibiotic treatment. There have been three postoperative deaths. One patient developed a fatal pulmonary embolism after open reduction and internal fixation of a femoral neck/shaft fracture combination and two patients with known widespread metastatic disease developed multisystem organ failure in the early postoperative period. One of these patients had an intraoperative fracture through one of the metastatic lesions. Furthermore, one patient with known renal dysfunction required postoperative dialysis, one patient survived a postoperative deep vein thrombosis, and one patient demonstrated a postoperative foot drop. No other major complications have been recorded among those patients who were captured in the hospital adverse event log.

One persistent deficit in the Hospital Lumiere system has been patient care coordination. Hospital staff and the providers attempt to help patients and families plan for discharge needs. Daily rounding and advancement of rehabilitation is coordinated. Postoperative care and rehabilitation, however, can be challenging due to the geography, transportation, infrastructure, and personal support of patients. Home therapy education is usually provided, but some patients are brought back to the ortho clinic for wound management, therapy sessions, and follow-up in coordination with either the local orthopaedic surgeon or the visiting teams.

Anecdotally, the HUP resident clinical skillset and also performance on standardized testing have improved through the course of this program. More detailed analysis with standardized testing or practical skills assessment has not yet been implemented but has been considered by the Haitian and American stakeholders. There is no analog to the Orthopaedic In-Training Examination in Haiti, and language barriers exist that currently preclude implementation of available metrics. However, the HUP program has grown each year. Graduates have remained in their native country as practicing orthopaedic surgeons, now with a growing peer network. Several of the graduates have gone on to receive fellowship training in other countries, which they have brought back to their country. Thus, we believe the SCOA resident training program has played a role in helping the HUP residents to develop the technical skills and self-directed learning necessary to succeed in an advanced training setting.

This success and the value of the learning experience at Lumiere have led to a proposal to have an upper level Haitian resident at Bonne Fin full-time under the supervision of the on-site staff surgeon, Dr. Oda, and the visiting SC teams. In addition, residents and faculty from the United States have reviewed and completed complex cases, prepared for culturally and spiritually sensitive behaviors, and properly followed Center for Disease Control guidelines for appropriate travel conduct, providing an excellent learning experience applied both at home and abroad.

## Discussion

Haiti is the poorest country in the western hemisphere. Its citizens' life experience is of third world conditions and austere medical care at best. A review of relevant literature reveals consistent recommendations for improving orthopaedic care in Haiti, calling for “continuous educational support for the new generation of residents”^1^ and noting “the objective of educational organizations should be to train local health-care workers at all levels in their own environment to provide sustainable and appropriate care so that the programs become self-sufficient and a continuous supply of competent personnel is ensured.”^2^ We agree with these recommendations and submit that the system that the SCOA Foundation has established serves these purposes. The SCOA Foundation's mission is to provide a unique training experience for the orthopaedic residents of SC through a coordinated service to the musculoskeletal needs of the Haitian people while collaboratively elevating the standard of orthopaedic training in Haiti. This mission is being accomplished as demonstrated by our results. Shultz et al^3^ have documented not only the interest of orthopaedic residents in international opportunities but also the barriers to these elective rotations. The SC residents are afforded the opportunity to collaborate with their fellow Haitian orthopaedic residents and participate in the care of the Haitian population.

ACGME guidelines require formal education of residents in five core competencies. The attending surgeons formally teach professionalism, patient care, medical knowledge, interpersonal and communication skills, and system-based practice during these rotations. Our residents and team members are mentored to provide culturally competent care in a resource-poor region. Several studies have demonstrated that participation in international electives plays a positive and influential role in the resident's education.^2,3^ Many of the SC residents have described this experience as “life changing” and the best rotation during their 5 years.

**Figure 3:**
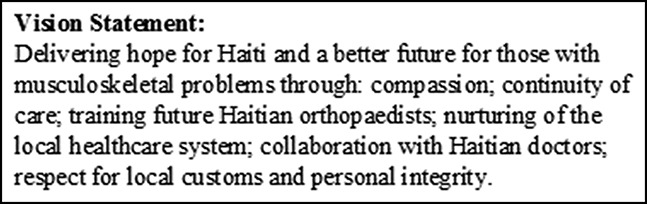
Vision statement.

The Haitian orthopaedic residents also recount the value of the exchange program. They receive structured didactic lectures from the visiting academic surgeons. They are supervised during clinical and surgical care at Hospital Lumiere by these surgeons and are able to operate and collaborate with their fellow US residents. They also have the opportunity to travel to SC and observe the residents and surgeons functioning within the ACGME framework. For some of them, it is their first opportunity to travel out of Haiti. They experience the dedication and commitment to academic excellence exhibited by the SC orthopaedic residents. The program director at Hospital de la Paix, Dr. Patrick Jumelle, has noticed improvements in the Haitian resident's dedication to reading and studying orthopaedic literature. They also demonstrate increased knowledge in the capacity of orthopaedic surgery to change lives, despite limited resources (Figure 3).

It deserves mention that the SCOA Foundation could not function without outside support and funding. Since taking on management of Hospital Lumiere, ACWR has provided outstanding support of the hospital infrastructure and staffing. They brought the hospital, and the community of Bonne Fin “back to life” after the earthquake by not only providing outstanding medical care to the local community in the mountains north of Les Cayes but also by providing economic opportunities to the surrounding area in a country that averages 14% unemployment. They have updated the power grid of the hospital with a state-of-the-art solar panel system and a new hydro-electric generator system, which has served to protect sensitive operating room equipment and provide more reliable power to the facilities. From the SCOA Foundation perspective, those individuals directly participating in the trips have provided much of the initial and ongoing funding. We have petitioned and received institutional support from the hospital systems of each residency program. We currently operate on a “bare bones” budget, which includes SCOA administrative support and Lindsey Demos, our volunteer coordinator. Given the initial success of the program, we feel it is imperative to broaden the scope of our funding and have therefore requested aid from local medical and nonmedical benefactors. Additional funding would certainly prove beneficial to the Foundation, allowing us to update on-site surgical equipment and provide travel scholarships for our Haitian orthopaedic residents.
